# Supplementary figures and images for: Horizontal gene transfer of molecular weapons can reshape bacterial competition
Source: PLoS Biol. 2025 May 21;23(5):e3003095. doi: 10.1371/journal.pbio.3003095 (PMC12094771; doi:10.1371/journal.pbio.3003095)

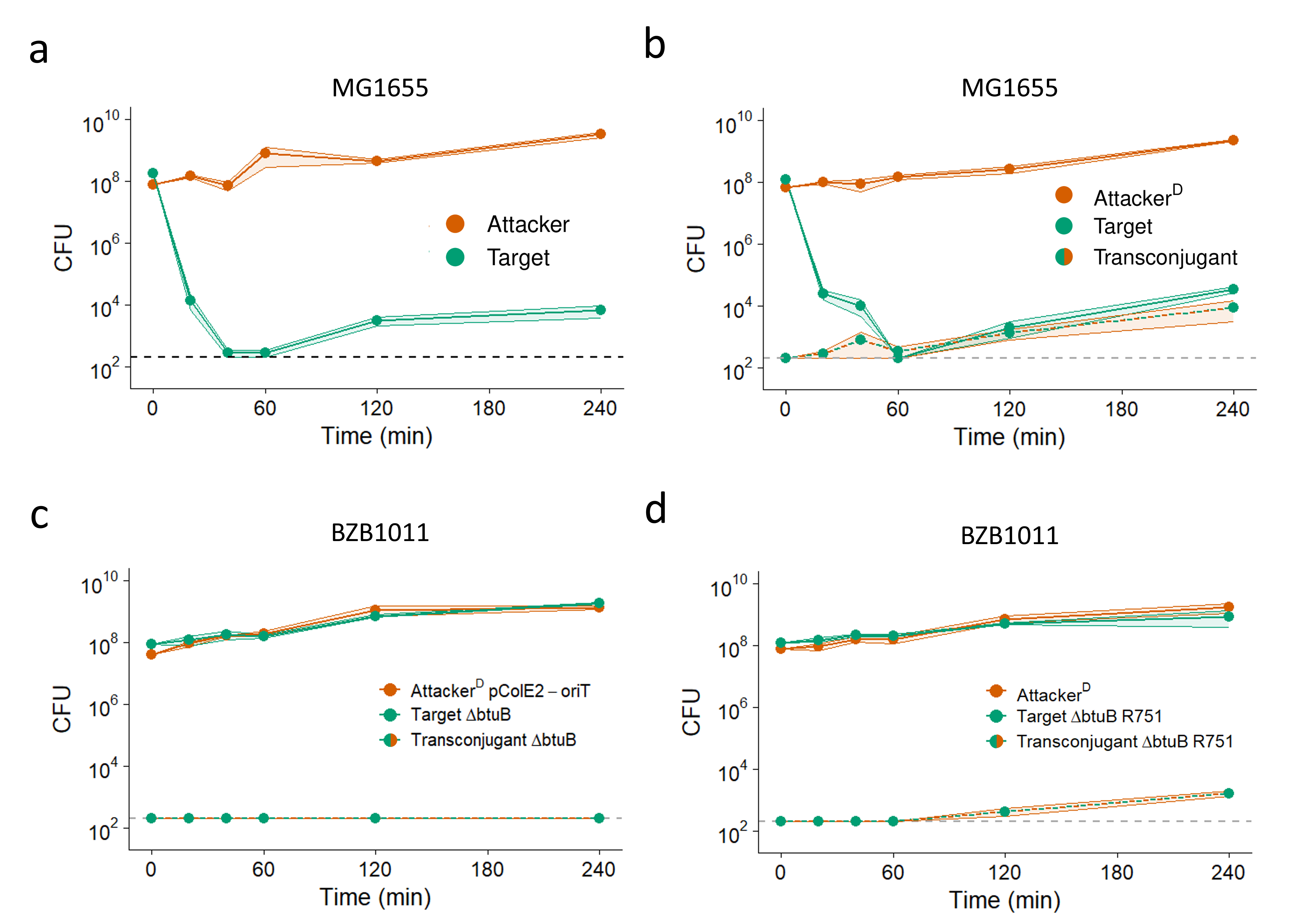

Supplement: S1 Fig — We conducted pairwise competition assays between different E. coli strains on LB agar plates. For each genotype, cell recovery (CFU) at each time point of co-culturing is shown. CFU for each time point after t = 0 were determined via destructive sampling of n = 3 independent replicates (see section “Materials and methods”). Means across replicates are shown as dots and connected by lines. Shaded ribbons around lines depict standard error across replicates. Dashed lines indicate the detection limit (200 CFU). (a) MG1,655-KmR pColE2-CmR (‘Attacker’) competed against MG1,655-GmR (‘Target’). No transconjugants (MG1,655-GmR pColE2-CmR) were detected. (b) MG1,655-KmR R751-SpR pColE2-CmR (‘AttackerD’) competed against MG1,655-GmR (‘Target’). ‘Transconjugant’ (MG1,655-GmR R751-SpR pColE2-CmR) CFU are shown as they emerge during the interaction. (c) BZB1011-KmR R751-SpR pColE2-oriT-AmpR (‘AttackerD pColE2-oriT’) competed against BZB1011-CmR ΔbtuB (‘Target ΔbtuB’). No transconjugants (BZB1011-CmR ΔbtuB pColE2-oriT-AmpR) were detected (‘Transconjugant ΔbtuB’). (d) BZB1011-KmR R751-SpR pColE2-AmpR (‘AttackerD’) competed against BZB1011-CmR ΔbtuB R751-SpR (‘Target ΔbtuB R751’). Transconjugant (BZB1011-CmR ΔbtuB R751-SpR pColE2-AmpR) CFU are shown as they emerge during the interaction (‘Transconjugant ΔbtuB R751’). Data underlying these figures is available from https://doi.org/10.5281/zenodo.10909492. (TIF) [file pbio.3003095.s001.tif]

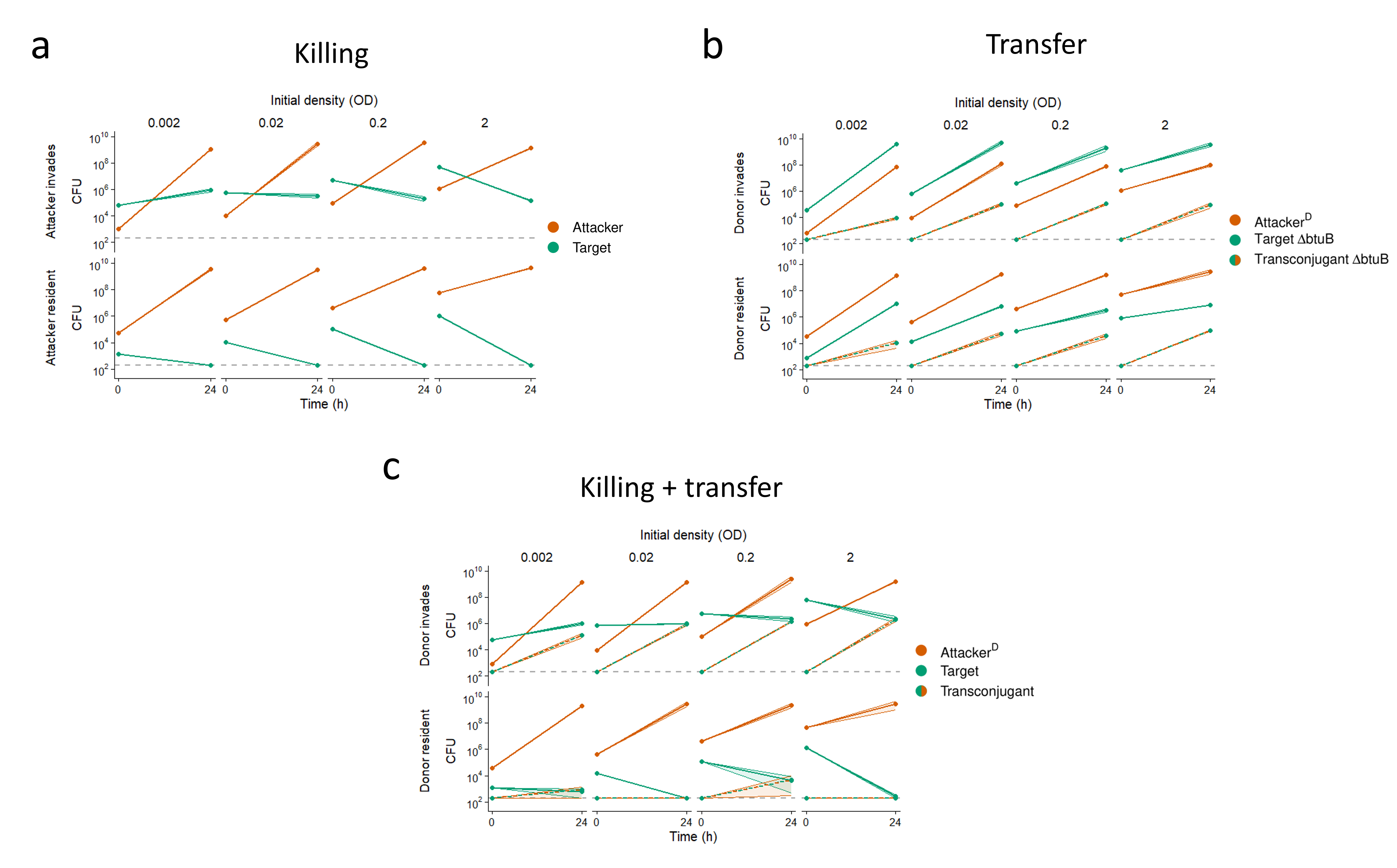

Supplement: S2 Fig — We conducted pairwise competition assays between different E. coli strains on LB agar plates. For each genotype, initial cell density and post-competition cell recovery (CFU) are shown. Competitions were initialized by adjusting preculture cell densities to an optical density (OD) of 0.002, 0.02, 0.2 or 2.0. Means across n = 3 replicates are shown as dots and connected by lines. Shaded ribbons around lines depict standard error across replicates. Grey dashed lines indicate the detection limit (200 CFU). Subsets of this dataset are shown in Fig 1d–f. (a) BZB1011-KmR pColE2-AmpR (‘Attacker’) competed against BZB1011-CmR (‘Target’). No transconjugants (BZB1011-CmR pColE2-AmpR) were detected. (b) BZB1011-KmR R751-SpR pColE2-AmpR (‘AttackerD’) competed against BZB1011-CmR ΔbtuB (‘Target ΔbtuB’). Transconjugant (BZB1011-CmR ΔbtuB R751-SpR pColE2-AmpR) CFU are shown as they emerge during the interaction (‘Transconjugant ΔbtuB’). (c) BZB1011-KmR R751-SpR pColE2-AmpR (‘AttackerD’) competed against BZB1011-CmR (‘Target’). ‘Transconjugant’ (BZB1011-CmR R751-SpR pColE2-AmpR) CFU are shown as they emerge during the interaction. Data underlying these figures is available from https://doi.org/10.5281/zenodo.10909492. (TIF) [file pbio.3003095.s002.tif]

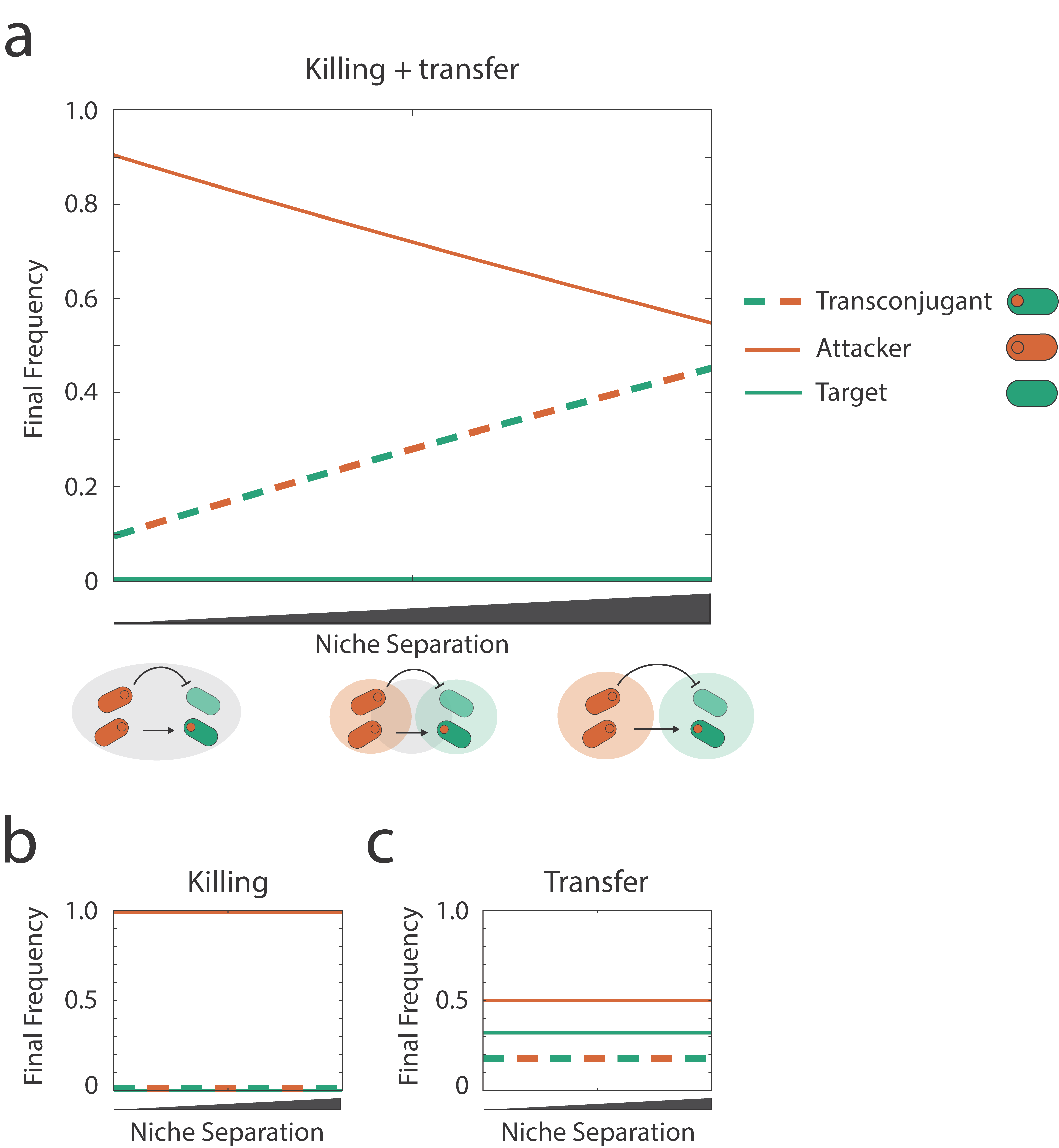

Supplement: S3 Fig — Using a constant initial pool of nutrients (N1 + N2 + N3 = 3.0), we observe the impact of varying levels of niche separation (i.e., metabolic diversity) on final strain frequencies. (a) In a scenario with both killing and transfer, the final frequency of the transconjugants is lowest when niche separation is lowest (left side of plot; N1 = 3.0; N2 + N3 = 0). As niche separation increases (N1 = decreasing; N2 + N3 = increasing), the final frequency of transconjugants increases and the final frequency of attackers decreases. Maximum transconjugant frequency is observed with complete niche separation (right side of plot; N1 = 0; N2 = N3 = 1.5). (b) In a scenario with only killing, attackers dominate across all conditions. (c) In a scenario with only transfer, final strain frequencies are constant regardless of niche separation. Across all conditions, N2 = N3. All parameters are default (Table 1) unless stated. Code and data underlying these figures are available from https://doi.org/10.5281/zenodo.14910561. (TIF) [file pbio.3003095.s003.tif]

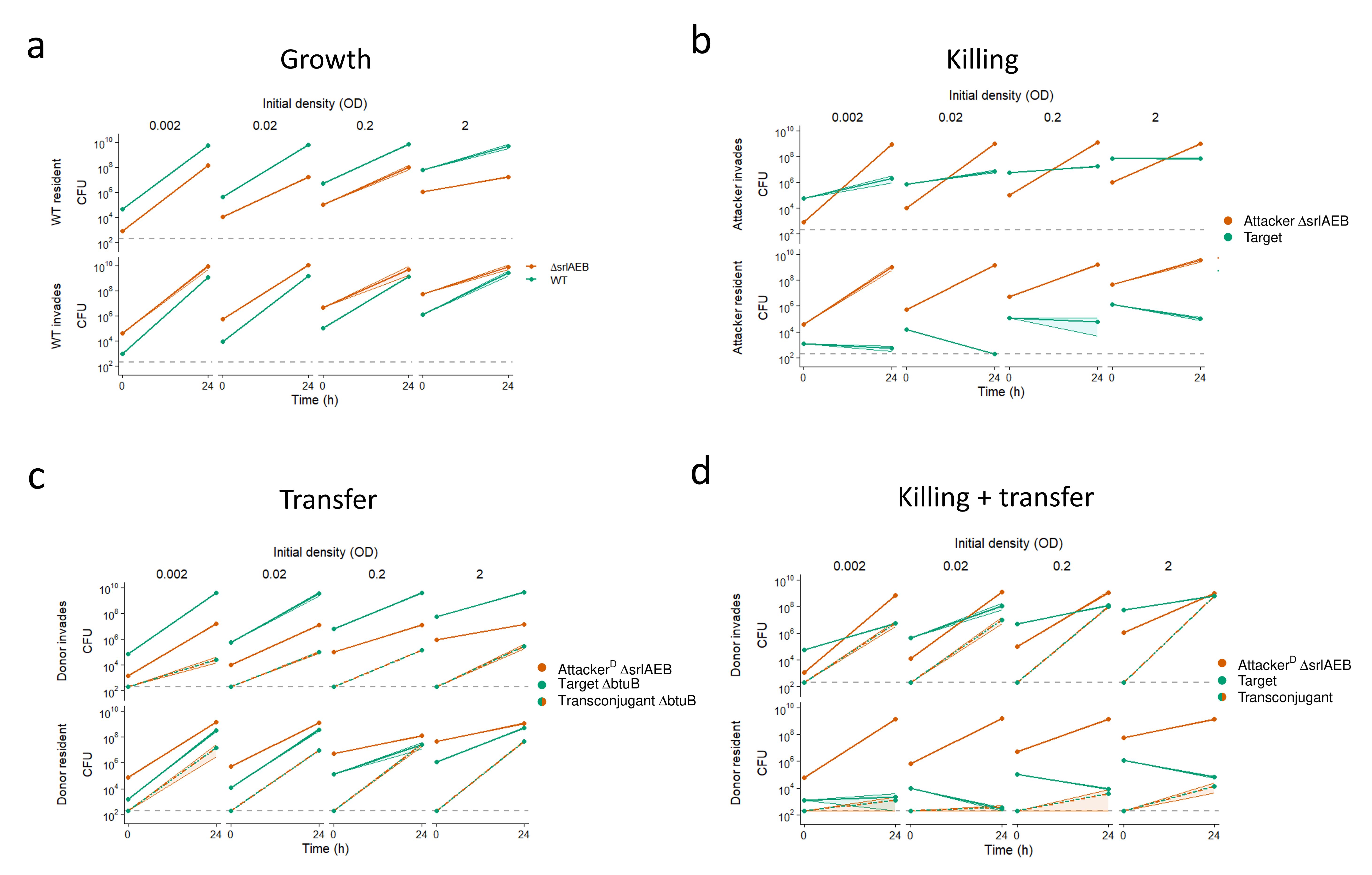

Supplement: S4 Fig — We conducted pairwise competition assays between different E. coli strains on LB agar plates. For each genotype, initial cell density and post-competition cell recovery (CFU) are shown. Competitions were initialized by adjusting preculture cell densities to an optical density (OD) of 0.002, 0.02, 0.2 or 2.0. Means across n = 3 replicates are shown as dots and connected by lines. Shaded ribbons around lines depict standard error across replicates. Grey dashed lines indicate the detection limit (200 CFU). Subsets of this dataset are shown in Fig 4a–c. (a) BZB1011-KmR ΔsrlAEB (‘ΔsrlAEB’) competed against BZB1011-CmR (‘WT’). (b) BZB1011-KmR ΔsrlAEB pColE2-AmpR (‘Attacker ΔsrlAEB’) competed against BZB1011-CmR (‘Target’). No transconjugants (BZB1011-CmR pColE2-AmpR) were detected. (c) BZB1011-KmR ΔsrlAEB R751-SpR pColE2-AmpR (‘AttackerD ΔsrlAEB’) competed against BZB1011-CmR ΔbtuB (‘Target ΔbtuB’). Transconjugant (BZB1011-CmR ΔbtuB R751-SpR pColE2-AmpR) CFU are shown as they emerge during the interaction (‘Transconjugant ΔbtuB’). (d) BZB1011-KmR ΔsrlAEB R751-SpR pColE2-AmpR (‘AttackerD ΔsrlAEB’) competed against BZB1011-CmR (‘Target’). ‘Transconjugant’ (BZB1011-CmR R751-SpR pColE2-AmpR) CFU are shown as they emerge during the interaction. Data underlying these figures is available from https://doi.org/10.5281/zenodo.10909492. (TIF) [file pbio.3003095.s004.tif]

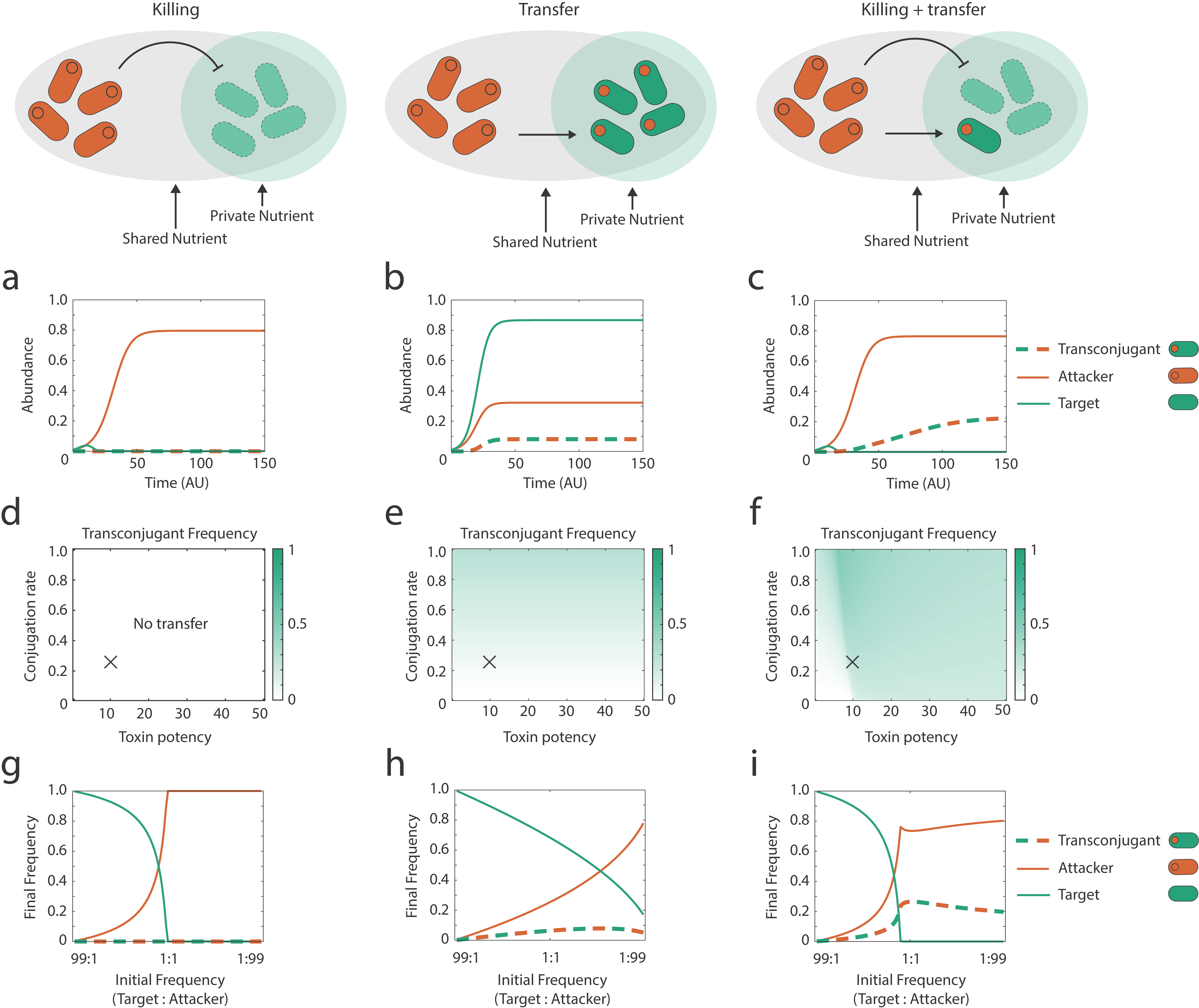

Supplement: S5 Fig — Modeling scenarios for each column are shown across the top row. (a–c) Example dynamics of the strains (attacker, target, transconjugant) during a contest using parameters that corresponding to the cross (X) shown in the parameter sweeps directly below (d–f) Transconjugant frequency at steady state (see section “Materials and methods”) in competitions as a function of conjugation rate (b) and toxin killing efficiency (E). (g–i) Final frequency of different strain types at steady state as a function of initial frequency of target and attacker strains. In the two-nutrient model, N3 = 0.25 in order to keep maximum observed growth rates similar for all strains. All other parameters are default (Table 1) unless stated. Code and data underlying these figures are available from https://doi.org/10.5281/zenodo.14910561. (TIF) [file pbio.3003095.s005.tif]

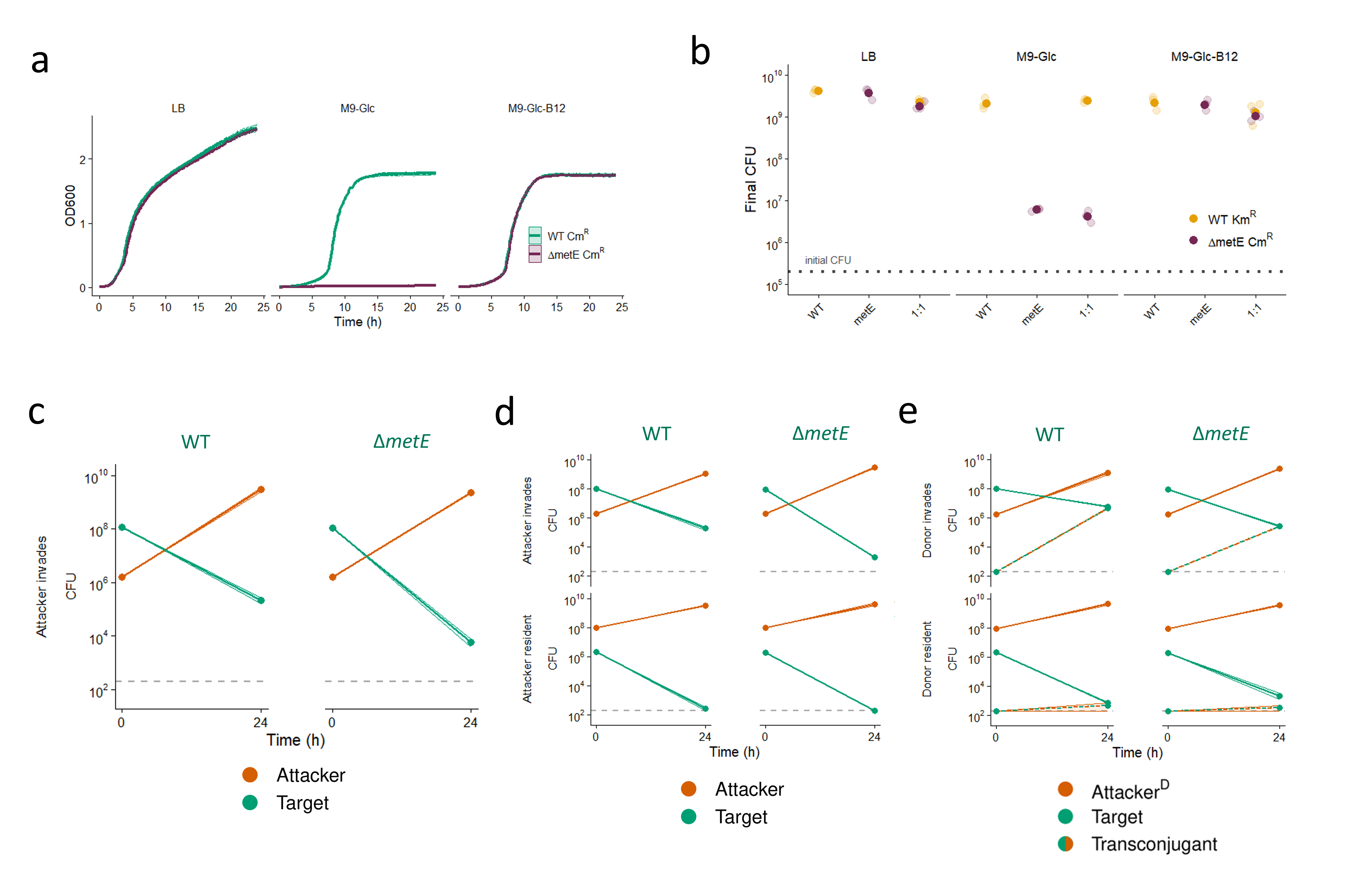

Supplement: S6 Fig — (a) Growth curves of BZB1011-CmR (‘WT CmR’) and ΔmetE (‘ΔmetE CmR’) in different nutrient media (LB, M9-Glc and M9-Glc-B12). Mean OD across n = 3 replicates are depicted as dots and connected by lines. Shaded ribbons around lines represent standard error across replicates. (b) BZB1011-KmR (‘WT KmR’) and BZB1011-CmR ΔmetE (‘ΔmetE CmR’) were grown for 24 h in either mono- or mixed cultures on different nutrient medium agar plates (LB, M9-Glc or M9-Glc-B12). Dotted line indicates initial cell density for all strains. Final CFU for n = 3 replicates and their means are shown as faint and solid color dots, respectively. (c–e) Pairwise competition assays on minimal medium agar plates. Initial cell density and post-competition cell recovery (CFU) for each genotype are shown. Means across n = 3 independent replicates are depicted as dots and connected by lines. For ‘Target’ data depicted in panel d (top left), n = 2. Shaded ribbons around lines depict standard error across replicates. Grey dashed lines indicate the detection limit (200 CFU). To test for differences in target survival, we used two-sided, two-sample t-tests on log-transformed CFU counts. (c; left) versus (c; right): t = −10.05, df = 4, p < 0.001. (e; top left) versus (e; top right): t = −22.64, df = 4, p < 0.001. (c+d) BZB1011-KmR pColE2-AmpR (‘Attacker’) competed against either BZB1011-CmR or BZB1011-CmR ΔmetE as ‘Target’. Panel c shows data from a pilot experiment used for sequencing spontaneously resistant clones (see section “Materials and methods”). (e) BZB1011-KmR R751-SpR pColE2-AmpR (‘AttackerD’) competed against BZB1011-CmR (Target). ‘Transconjugant’ (BZB1011-CmR (ΔmetE) R751-SpR pColE2-AmpR) CFU are shown as they emerge during the interaction. Data underlying these figures is available from https://doi.org/10.5281/zenodo.10909492. (TIF) [file pbio.3003095.s006.tif]

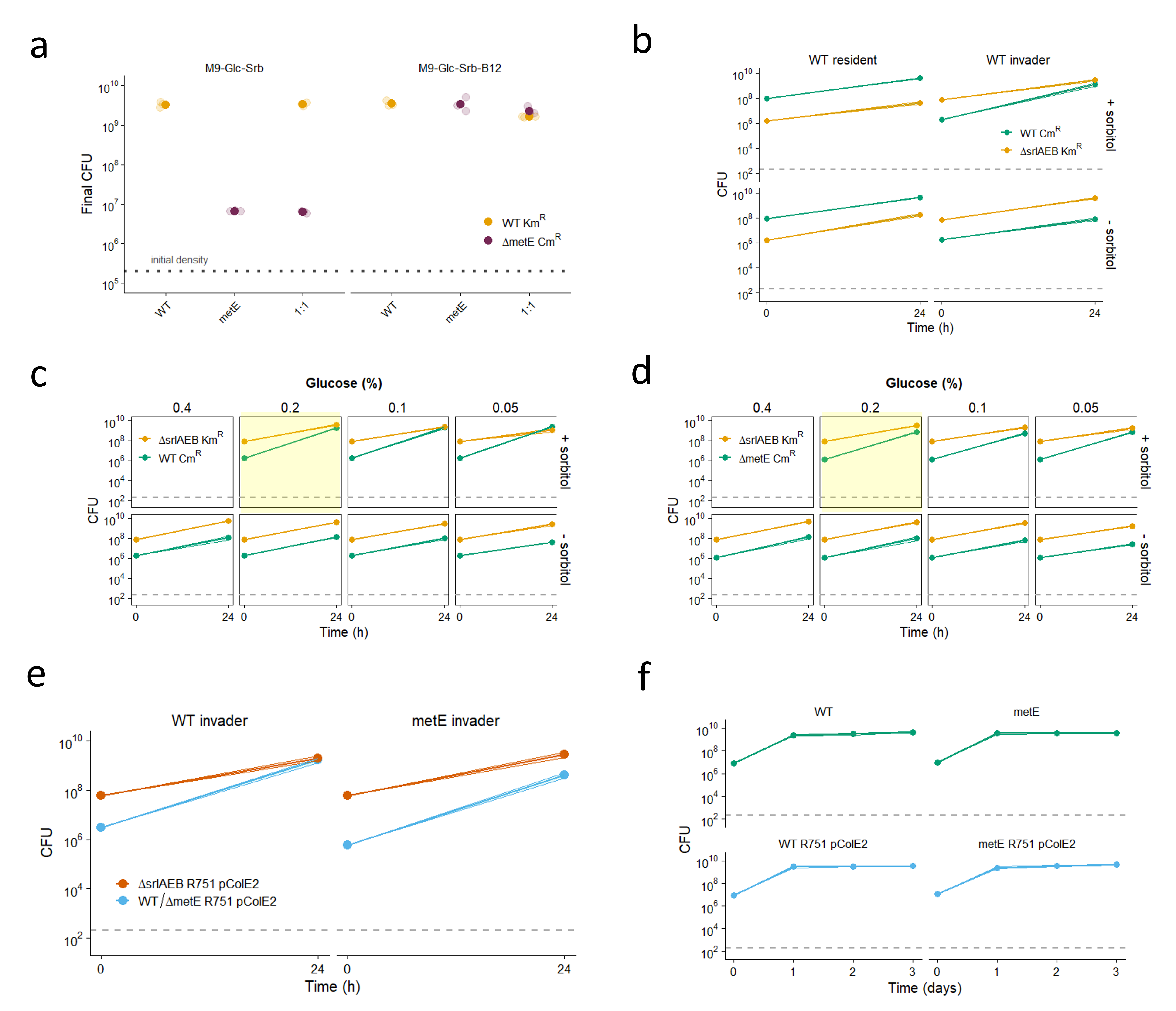

Supplement: S7 Fig — (a) BZB1011-KmR (‘WT KmR’) and BZB1011-CmR ΔmetE (‘ΔmetE CmR’) were grown for 24 h in mono- and mixed cultures on minimal medium agar plates supplemented with glucose and sorbitol, either with (M9-Glc-Srb-B12) or without vitamin B12 (M9-Glc-Srb). Dotted line indicates initial cell density for all strains. Final CFU for n = 3 replicates and their means are shown as faint and solid color dots, respectively. (b–d) BZB1011-KmR ΔsrlAEB (‘ΔsrlAEB KmR’) was competed either against (b, c) BZB1011-CmR (‘WT CmR’); or (d) BZB1011-CmR ΔmetE (‘ΔmetE CmR’) for 24 h on minimal medium agar plates, with or without the addition of sorbitol. For each strain, initial cell density and post-competition cell recovery (CFU) are shown. Means across n = 3 replicates are depicted as dots and connected by lines. Shaded ribbons around lines represent standard error across replicates. Grey dashed lines indicate the detection limit (200 CFU). Glucose concentrations in minimal medium were either the standard 0.2% (b) or were experimentally varied from 0.05% to 0.4% (c and d). Yellow highlight indicates standard media conditions used in experiments shown in Fig 5 and (a), (b), (e) and (f). (e) BZB1011-KmR ΔsrlAEB R751-SpR pColE2-AmpR (‘ΔsrlAEB R751 pColE2) was competed either against BZB1011-CmR R751-SpR pColE2-AmpR or against BZB1011-CmR ΔmetE R751-SpR pColE2-AmpR for 24 h on minimal medium agar supplemented with sorbitol and vitamin B12 (M9-Glc-Srb-B12). For each strain, initial cell density and post-competition cell recovery (CFU) are shown. Means across n = 3 replicates are depicted as dots and connected by lines. Shaded ribbons around lines represent standard error across replicates. Grey dashed lines indicate the detection limit (200 CFU). (f) BZB1011-CmR (‘WT’) and BZB1011-CmR ΔmetE (‘metE‘), either with or without carrying plasmids R751-SpR and pColE2-AmpR, were serially passaged in monocultures over 3 days. CFU were determined each day before transferring to fresh nutrient plates (see sect [file pbio.3003095.s007.tif]

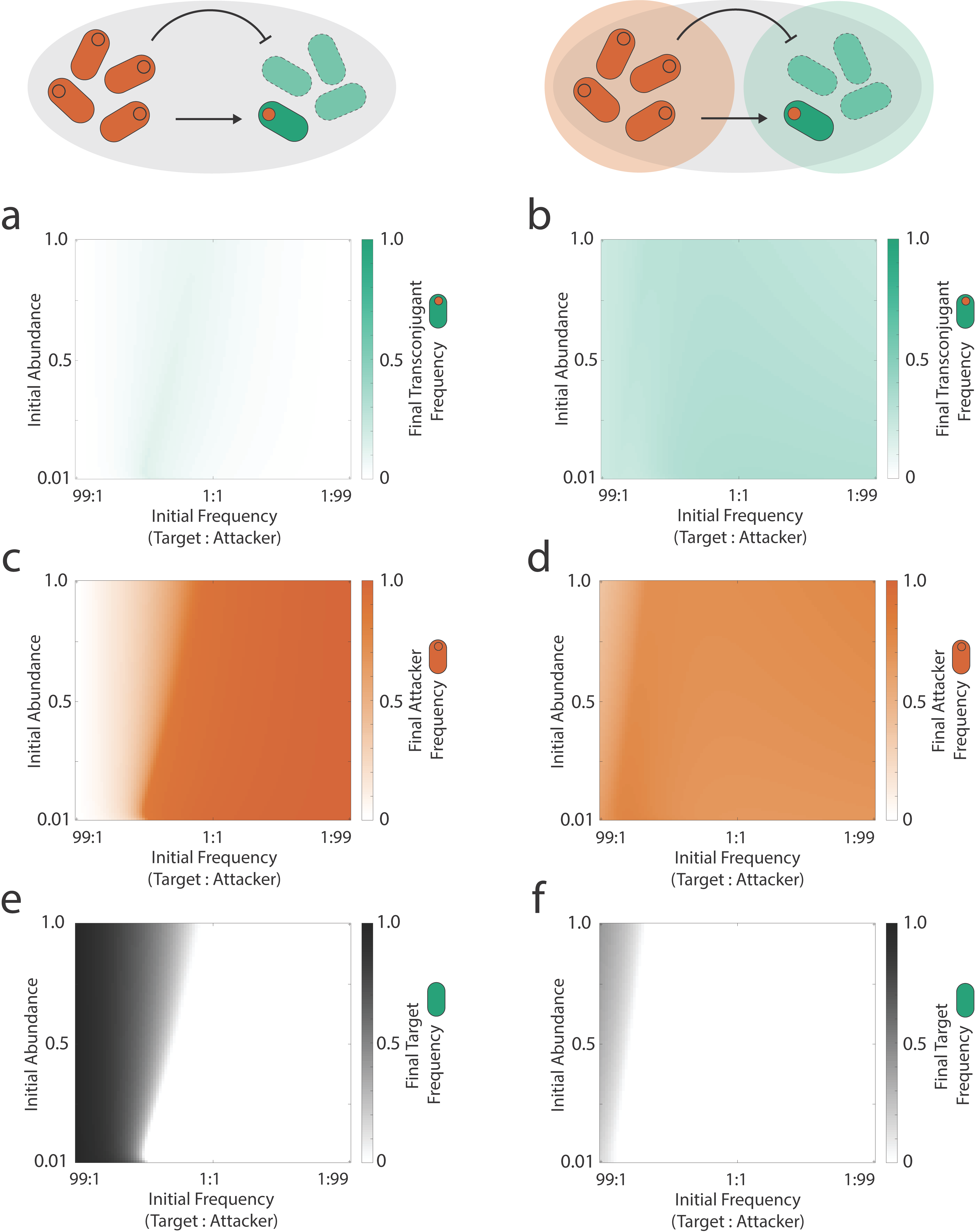

Supplement: S8 Fig — Each heat map ranges from initial population density of 0.01 to 1.0, and varies the initial frequency of the target and attacker strains from 99:1 to 1:99. Scenarios of complete niche overlap (a, c, e) and metabolic diversity (b, d, f) are represented. All parameters are default unless otherwise noted. Code and data underlying these figures are available from https://doi.org/10.5281/zenodo.14910561. (TIF) [file pbio.3003095.s008.tif]
