# Supplementary material for: Horizontal gene transfer of molecular weapons can reshape bacterial competition
Source: PLoS Biol. 2025 May 21;23(5):e3003095. doi: 10.1371/journal.pbio.3003095 (PMC12094771; doi:10.1371/journal.pbio.3003095)
Supplement: S1 Table — (DOCX) [file pbio.3003095.s010.docx]

**Table S1. Resistance phenotypes.**

| **Figure** | **Subpanel** | **n (target clones)** | **n (target clones, resistant)^[[1]](#footnote-1)^** | **n (transconjugant clones)** | **n (transconjugant clones, resistant)** |
| --- | --- | --- | --- | --- | --- |
| 2A | - | 15^[[2]](#footnote-2)^ | 14 (93%) | - | - |
| 2C^[[3]](#footnote-3)^ | - | 30^†^ | 26 (87%) | 15^†^ | 5 (33%) |
| 4D | top | 9 | 9 (100%) | - | - |
| 4D | bottom | 6 | 6 (100%) | - | - |
| 4E | top | 9 | 0 (0%) | 9 | 0 (0%) |
| 4E | bottom^[[4]](#footnote-4)^ | 6 | 0 (0%) | 6 | 0 (0%) |
| 4E | bottom^[[5]](#footnote-5)^ | 3 | 3 (100%) | - | - |
| 5A | top left | 3 | 3 (100%) | - | - |
| 5A | top right | 3 | 0 (0%) | - | - |
| 5B | top left | 3 | 0 (0%) | 3 | 0 (0%) |
| 5B | top right | 3 | 0 (0%) | 3 | 0 (0%) |
| 5B | bottom left | 3 | 1 (33%) | 3 | 0 (0%) |
| 5B | bottom right | 3 | 0 (0%) | 3 | 0 (0%) |
| 5C | top | 6 | 0 (0%)^[[6]](#footnote-6)^ | - | - |
| 5D | top | 3 | 0 (0%) | 3 | 0 (0%) |
| 5D | bottom | 3 | 0 (0%) | 3 | 0 (0%) |
| S1A | - | 15^†^ | 12 (80%) | - | - |
| S1B | - | 13^†^ | 0 (0%) | 14^†^ | 0 (0%) |
| S6C | left | 8 | 8 (100%) | - | - |
| S6C | right | 8 | 3 (38%) | - | - |

1. “resistant” = colicin resistance profile suggests *btuB* mutation (see Methods) [↑](#footnote-ref-1)
2. across all time points [↑](#footnote-ref-2)
3. across two independent experiments [↑](#footnote-ref-3)
4. two replicates [↑](#footnote-ref-4)
5. third replicate [↑](#footnote-ref-5)
6. 6/6 multi-colicin resistant, but not consistent with *btuB* mutation; possibly *tolB* mutants (see Methods) [↑](#footnote-ref-6)
