# Supplementary material for: Horizontal gene transfer of molecular weapons can reshape bacterial competition
Source: PLoS Biol. 2025 May 21;23(5):e3003095. doi: 10.1371/journal.pbio.3003095 (PMC12094771; doi:10.1371/journal.pbio.3003095)
Supplement: S2 Table — (DOCX) [file pbio.3003095.s011.docx]

**Table S2. Strains and plasmids used in this study.**

| **STRAINS** | **Name/Label** | **Species** | **Genotype** | **Source** |
| --- | --- | --- | --- | --- |
|  | BZB1011 | *Escherichia coli* | λ-, *gyrA586*(Nal^R^), IN(*rrnDrrnE*)1, *rpsL*-(Str^R^), *rph*-1 | (1) |
|  | WT Km^R^ | *Escherichia coli* | BZB1011 *yidX*-aph(3')-II-*yidA*(Km^R^) | Erik Bakkeren |
|  | WT Cm^R^ | *Escherichia coli* | BZB1011 *yidX*-*cat*-*yidA*(Cm^R^) | Erik Bakkeren |
|  | MG1655 | *Escherichia coli* | F- λ- *ilvG*- *rfb*-50 *rph*-1 | Colin Kleanthous |
|  | MG1655 Km^R^ | *Escherichia coli* | MG1655 *attTn7*::aph(3')-II(Km^R^) | This study |
|  | MG1655 Gm^R^ | *Escherichia coli* | MG1655 Gm^R^ | (2) |
|  | ΔmetE | *Escherichia coli* | BZB1011 Δ*metE*::*cat*(Cm^R^) | This study |
|  | ΔsrlAEB | *Escherichia coli* | BZB1011 Δ*srlAEB::* aph(3')-II(Km^R^) | Erik Bakkeren |
|  | Attacker | *Escherichia coli* | BZB1011 *yidX*-aph(3')-II-*yidA*(Km^R^) pColE2-Amp^R^ | This study |
|  | Attacker^D^ | *Escherichia coli* | BZB1011 *yidX*-aph(3')-II-*yidA*(Km^R^) R751-Sp^R^ pColE2-Amp^R^ | This study |
|  | Target | *Escherichia coli* | BZB1011 *yidX*-*cat*-*yidA*(Cm^R^) | Erik Bakkeren |
|  | Target ΔbtuB | *Escherichia coli* | BZB1011 *yidX*-*cat*-*yidA*(Cm^R^) Δ*btuB* | This study |
|  | Attacker ΔsrlAEB | *Escherichia coli* | BZB1011 Δ*srlAEB::*aph(3')-II(Km^R^) pColE2-Amp^R^ | This study |
|  | Attacker^D^ ΔsrlAEB | *Escherichia coli* | BZB1011 Δ*srlAEB::*aph(3')-II(Km^R^) R751-Sp^R^ pColE2-Amp^R^ | This study |
|  | Attacker (MG1655) | *Escherichia coli* | MG1655 *attTn7*::aph(3')-II(Km^R^) pColE2-Cm^R^ | This study |
|  | Attacker^D^ (MG1655) | *Escherichia coli* | MG1655 *attTn7*::aph(3')-II(Km^R^) R751-Sp^R^ pColE2-Cm^R^ | This study |
|  | Attacker^D^ pColE2-oriT | *Escherichia coli* | BZB1011 *yidX*-aph(3')-II-*yidA*(Km^R^) R751-Sp^R^ pColE2-oriT-Amp^R^ | This study |
|  | JKe201 | *Escherichia coli* | MFDpir Δ*mcrA*  Δ(*mrr-hsdRMS-mcrBC*)  *aac(3)IV::lacIq* | (3) |
|  | JKe201 pTNS2 | *Escherichia coli* | JKe201 pTNS2 | Sean Booth |
|  | JKe201 pColE2-Amp^R^ | *Escherichia coli* | JKe201 pColE2-Amp^R^ | This study |

|  | BZB1011 pKD46 | *Escherichia coli* | BZB1011 pKD46 | Erik Bakkeren |
| --- | --- | --- | --- | --- |
|  | One Shot^TM^ TOP10 | *Escherichia coli* | F^–^mcrA Δ(mrr-hsdRMS-mcrBC) φ80lacZΔM15 ΔlacX74 recA1 araD139 Δ(ara-leu)7697 galU galK λ–rpsL(Str^R^) endA1 nupG | Invitrogen |
|  | TOP10 R751-Sp^R^ | *Escherichia coli* | One Shot^TM^ TOP10 R751-Sp^R^ | This study |
| **PLASMIDS** | pColE2 |  | pColE2-P9 | (4) |
|  | pColE2-Amp^R^ |  | pColE2-P9-Amp^R^ | Erik Bakkeren |
|  | pColE2-Cm^R^ |  | pColE2-P9-Cm^R^ | (5) |
|  | pColE2-ΔoriT |  | pColE2-ΔoriT-Amp^R^ | This study |
|  | R751 |  | R751-Sp^R^ | (6) |
|  | pUC18R6KT-mini-Tn7T-Km |  | pUC18R6KT-mini-tn7-Km (Addgene plasmid #64969) | (7) |
|  | pTNS2 |  | pTNS2 | (7) |
|  | pTML8 |  | pTML8 | (8) |
|  | pKD3 |  | pKD3 | (9) |
|  | pKD4 |  | pKD3 | (9) |
|  | pKD46 |  | pKD46 | (9) |
|  | pUC19 |  | pUC19 | New England Biolabs |

**REFERENCES**

1. Pugsley AP, Schwartz M. A genetic approach to the study of mitomycin-induced lysis of Escherichia coli K-12 strains which produce colicin E2. MGG Mol Gen Genet. 1983 Jun;190(3):366–72.

2. Basler M, Ho BT, Mekalanos JJ. Tit-for-tat: Type VI secretion system counterattack during bacterial cell-cell interactions. Cell. 2013 Feb 14;152(4):884–94.

3. Harms A, Liesch M, Körner J, Québatte M, Engel P, Dehio C. A bacterial toxin-antitoxin module is the origin of inter-bacterial and inter-kingdom effectors of Bartonella. PLOS Genet. 2017 Oct 26;13(10):e1007077.

4. Pugsley AP. Escherichia coli K12 strains for use in the identification and characterization of colicins. J Gen Microbiol. 1985;131(2):369–76.

5. Mavridou DAI, Gonzalez D, Kim W, West SA, Foster KR. Bacteria use collective behavior to generate diverse combat strategies. Curr Biol. 2018 Feb 5;28(3):345-355.e4.

6. Bains A, Wilson JW. Differentially Marked IncP-1β R751 Plasmids for Cloning via Recombineering and Conjugation. Pol J Microbiol. 2019 Dec;68(4):559–63.

7. Choi KH, Gaynor JB, White KG, Lopez C, Bosio CM, Karkhoff-Schweizer RR, et al. A Tn7-based broad-range bacterial cloning and expression system. Nat Methods. 2005 Jun;2(6):443–8.

8. Krishna Kumar R, Meiller-Legrand TA, Alcinesio A, Gonzalez D, Mavridou DAI, Meacock OJ, et al. Droplet printing reveals the importance of micron-scale structure for bacterial ecology. Nat Commun. 2021 Feb 8;12(1):857.

9. Datsenko KA, Wanner BL. One-step inactivation of chromosomal genes in Escherichia coli K-12 using PCR products. Proc Natl Acad Sci. 2000 Jun 6;97(12):6640–5.
