# Supplementary material for: Horizontal gene transfer of molecular weapons can reshape bacterial competition
Source: PLoS Biol. 2025 May 21;23(5):e3003095. doi: 10.1371/journal.pbio.3003095 (PMC12094771; doi:10.1371/journal.pbio.3003095)
Supplement: S3 Table — (DOCX) [file pbio.3003095.s012.docx]

**Table S3. Primers used in this study.**

| **Primer name** | **Sequence (5′-3′)** | **Purpose** | **Source** |
| --- | --- | --- | --- |
| TML-P9 | ATAGCAGGGAAACCACCGCC | verification of *btuB* deletion | (1) |
| TML-P10 | GCAGATTTTGCATCCGGGGC | verification of *btuB* deletion | (1) |
| metE_del_fw | ATGACAATATTGAATCACACCCTCGGTTTCCCTCGCGTTGATGGGAATTAGCCATGGTCC | PCR for *metE* deletion | This study |
| metE_del_rv | CCCCGACGCAAGTTCTGCGCCGCCTGCACCATGTTCGCCATGTAGGCTGGAGCTGCTTC | PCR for *metE* deletion | This study |
| metE_ver_up | GAGCTATCATGCCGCATCTG | verification of *metE* deletion | This study |
| metE_ver_dw | GTTGGCTGCGTTTCTCCAC | verification of *metE* deletion | This study |
| yidX-yidA_CmKan_For | GGGCGGGCAAACAGCATAAACGCGTTTGCCCGCTTACTGATGTAGGCTGGAGCTGCTTC | PCR for Km^R^ or Cm^R^ insertion at neutral locus | This study |
| yidX-yidA_CmKan_Rev | ACCGCTGCAATTTCTGGTTGTATATGCAGTAAACCAATAAATGGGAATTAGCCATGGTCC | PCR for Km^R^ or Cm^R^ insertion at neutral locus | This study |
| yidX-yidA_ver_up | CTTCAGTGAAAGAAGTGGC | verification of Km^R^ or Cm^R^ insertion at neutral locus | This study |
| yidX-yidA_ver_dw | CTATTCACCCAGAGGCATTC | verification of Km^R^ or Cm^R^ insertion at neutral locus | This study |
| srlAEB_del_for | CCGTTTGGTAATAAAACAATAAATCCTGAAGGAGAGAACATGTAGGCTGGAGCTGCTTC | PCR for Km^R^ insertion at srlAEB locus | This study |
| srlAEB_del_Rev | TTTGCCCACCACCGATGACAACGGCAACCTGATTCATTTTATGGGAATTAGCCATGGTCC | PCR for Km^R^ insertion at srlAEB locus | This study |
| srlAEB_ver_up | CTGATTAGATTAGGTTGCCG | verification of Km^R^ insertion at srlAEB locus | This study |
| srlAEB_ver_dw | GAATATCGACAACCGCGAC | verification of Km^R^ insertion at srlAEB locus | This study |
| amp_fw | GAGTGTTCTGCTCATCGCGGAACCCCTATTTGTTTATTTTTCT | amplification of AmpR fragment from pUC19 | This study |
| amp_rv | ATGATAAATCGCCATGAAGATCCTTT | amplification of AmpR | This study |

|  | GATCTTTTCTACGGGGTCT | fragment from pUC19 |  |
| --- | --- | --- | --- |
| e2_amp_fw | GATCAAAGGATCTTCATGGCGATTTATCATCTCAGCATGAAAA | deletion of oriT from pColE2-P9 | This study |
| e2_amp_rv | AATAGGGGTTCCGCGATGAGCAGAACACTCGAACAGAAGAT | deletion of oriT from pColE2-P9 | This study |
| e2_1 | AGACCTGGCATGAGTGGAAG | sequence verification of pColE2-ΔoriT-Amp^R^ | This study |
| e2_2 | ACGGCATCAATTCCAGGTGC | sequence verification of pColE2-ΔoriT-Amp^R^ | This study |
| e2_3 | ACCAATCAGTCAGGATGGTGG TG | sequence verification of pColE2-ΔoriT-Amp^R^ | This study |
| e2_4 | CACCGCCAAGATTGATCACG | sequence verification of pColE2-ΔoriT-Amp^R^ | This study |
| e2_5 | AGTCGAGCGACGTACTACCG | sequence verification of pColE2-ΔoriT-Amp^R^ | This study |
| e2_6 | GATGTGGCGTTTCATCACATGG | sequence verification of pColE2-ΔoriT-Amp^R^ | This study |
| e2_7 | CCGTGAAACGGCATTAGTCG | sequence verification of pColE2-ΔoriT-Amp^R^ | This study |
| ampR_fw | GCTGGCTGGTTTATTGCTG | sequence verification of pColE2-ΔoriT-Amp^R^ | This study |
| Out_pColE2_For | GCATAGTTATGCAACGCGC | insertion of Amp^R^ into pColE2-P9 | This study |
| Out_pColE2_Rev | ACGAAAGCGATGCGCGATC | insertion of Amp^R^ into pColE2-P9 | This study |
| GibsColE2_Amp_For | GTGTTCAGAACGCACGAAACCGATCGCGCATCGCTTTCGTCACCGTCATCACCGAAACG | insertion of Amp^R^ into pColE2-P9 | This study |
| GibsColE2_Amp_Rev | AATGCGTCAGAATCGTTTTTAGCGCGTTGCATAACTATGCCTGACGCTCAGTGGA ACG | insertion of Amp^R^ into pColE2-P9 | This study |
| BtuB_BZB.F | CTGAAATATGGGGTGGATGCTTTACAATGATTAAAAAAGCTTCG | *btuB* sequencing | This study |
| BtuB_BZB.R | CCTGCAATGCATATCAGAAGGTGTAGCTGCCAG | *btuB* sequencing | This study |

**REFERENCES**

1. Krishna Kumar R, Meiller-Legrand TA, Alcinesio A, Gonzalez D, Mavridou DAI, Meacock OJ, et al. Droplet printing reveals the importance of micron-scale structure for bacterial ecology. Nat Commun. 2021 Feb 8;12(1):857.
