# Supplementary material for: Horizontal gene transfer of molecular weapons can reshape bacterial competition
Source: PLoS Biol. 2025 May 21;23(5):e3003095. doi: 10.1371/journal.pbio.3003095 (PMC12094771; doi:10.1371/journal.pbio.3003095)
Supplement: Attachment — Submitted filename: Response.pdf [file pbio.3003095.s015.pdf]

Reviewer #1: Ellie Harrison

Reviewer #1: This is a really nicely implemented dissection of the impact of mobility on bacterial 'weapons' involved in competition. The authors combine modelling and experiments to break down the impacts of gene mobility for competition in a really clear and readable way. They show clearly that the hypothesis that HGT to the target of competition will not always reduce the effectiveness of the competition (which is what we often see for temperate phages where transmission is basically inherent to attack and competition often flames out quickly). They then show convincingly that in more nuanced and realistic communities the effect of transfer can have greater effects.

Response 1: We thank the reviewer for this very positive assessment.

I commend the authors here on their excellent work and have only one minor, somewhat philosophical point to make, which may or may not augment the discussion. As they state their work nicely supports the idea that the real benefactor of transfer of these gene cassettes is the mobile element. However the search for conditions which allow the recipients to out complete the donors might be less important when seen from the plasmid's eye view. Either way the plasmid wins in these short term competitions - but in the long term the bet hedging (I agree) is key. Therefore i would assume that the resource heterogeneity principle also works in a temporarily or spatially heterogenous environment.

Response 2: Thank you for raising this interesting point. We agree that the plasmid has a different interest to the bacterial strains, i.e. which strain wins in competition will typically be more important to the strains than a mobile plasmid. Given these plasmids are used in bacterial warfare, we wanted to consider the evolutionary interests of the bacteria as well as the plasmids. Indeed, we started this project very much focussed on the bacteria and we have, to some extent, tried to write the manuscript to reflect this journey from a bacterial focus to a plasmid one.

Reviewer #2:

The manuscript "Horizontal gene transfer can reshape bacterial warfare" by Granato et al. addresses a highly significant and thought-provoking question. Bacteria frequently encode toxins, such as bacteriocins. Cells harboring genes for these toxins gain a distinct advantage by eliminating nearby sensitive cells. However, a paradox arises: why are toxin genes predominantly located on conjugative or mobilizable plasmids rather than on the bacterial chromosome? This presents a conundrum, as plasmids carry the toxin and the corresponding immunity genes, which transfer to neighboring cells, rendering those cells immune to the toxin's effects.

The question posed is highly relevant. As the authors mention in the Discussion, in Escherichia coli, all colicins are plasmid-encoded rather than chromosomally encoded, a phenomenon for which no explanation has been provided until now. This issue is of particular significance to

Microbiology, where toxins are prevalent, and Evolutionary Biology, where genetic conflicts play a central role. Furthermore, in the context of health, this manuscript offers valuable insights into the optimal strategy for enhancing the success of toxin genes within a microbiome (for example).

Response 3: Many thanks for this positive evaluation.

The authors propose the following explanatory hypothesis: encoding toxin/immunity genes on plasmids (rather than on the chromosome) provides an advantage to these genes when the donor and potential recipient cells do not compete intensely for nutrients. The authors employ mathematical and computational modeling and experiments to test this hypothesis.

A) The manuscript is very clear, except for the Abstract. In my (modest) opinion, the abstract doesn't do justice to the aim (scientific question) and the work done. For example, the second sentence of the abstract is:"Genes encoding many of these weapons can be transmitted horizontally, but the impact on bacterial warfare is not understood.". What do you mean? Why is it not understood? What is missing? Consider, for example, these two sentences: "The horizontal mobility of toxin and immunity genes (…) presents a unique problem. If gene transfer happens during an encounter with a competitor, the target becomes immune to the toxin, rendering the bacterial weapon useless and negating any potential fitness benefits for the producer. ". These are very clear sentences in the Introduction (Lines 39-41): much clearer than those of the abstract because they clearly state the scientific problem. My suggestion is to write something like this in the Abstract.

Response 4: We thank the reviewer for these suggestions on how to improve the Abstract, and have revised it to be more explicit about the problem in the set up.

B) The experiments performed corroborate the hypothesis. However, contrary to the authors' claim, the computer/mathematical model could not "predict" the experimental results. Why? Because the authors made a model of a liquid (non-structured) environment, whereas the experiments were in plates. Interestingly, it is precisely with colicins that Lin Chao and Bruce Levin (1981, PNAS) have shown a strong difference between the two environments (regarding expected experimental outcomes). I do not think this is a problem for this manuscript. Still, I ask the authors to state this point clearly. That is, the verbal hypothesis is corroborated in the "liquid" simulations ("liquid" because they used ordinary differential equations) and in a structured environment. It should be clear that the author's aim is not to prove that the results of the experiments corroborate the computer model. It is to show that their hypothesis is corroborated by simulations of an unstructured environment and experiments in a structured environment. I suggest small changes in the text. For example, in line 101, one can read, "To test the predictions of our model empirically, we turned to a well-characterized family of antibacterial toxins: the colicins."; I suggest changing this to something like "To test our hypothesis empirically, we turned to a well-characterized family of antibacterial toxins: the colicins."

Response 5: We have adopted the phrasing you suggested and make it clearer that the model does not explicitly capture spatial structure beyond patch level structure as follows in the results:

"For simplicity, our model assumes that cells are well-mixed in a given patch. However, our experiments introduce a degree of local spatial structure by studying bacteria growing on agar. As we will show, our modelling is robust to this introduction of spatial structure with predictions that are well supported by the experiments." (L117-120)

C) In lines 233 - 235, one reads the following: "Note that this experimental design is slightly different to our second model, because only one of the two strains has a private nutrient, rather than both as in our modelling. However, we show in the supplement that this change does not affect our modelling predictions (Fig. S5)". I understand why it is equivalent, but why not do the modeling in that way: only one of the two strains has a private nutrient?

Response 6: Thank you for this comment. We also were unsure which version of the model made the most sense in the main text. We believe the model which uses private nutrients for both strains, along with a common pool of shared nutrients, probably best captures 'real' interactions between microbes in nature. Therefore, we decided to use this model as the main model in the text (Fig. 3). Importantly, the model which uses a private nutrient for just one strain shows very similar results (Fig. S5). So I suppose it is a balance of either leading with a model to capture what we expect in nature, or a model to capture how we conduct the experiments. We favour the former as it likely makes the model more generally applicable, but importantly, both models show very similar results.

D) Statistics: The authors should apply Shapiro-Wilk tests (or others) to ensure the normality of the data before performing the t-test (or use a non-parametric test if the data are not normal).

We did apply the Shapiro-Wilk test to ensure normality of the data before performing t-tests, but failed to mention this in the relevant methods sections. Apologies for the omission, this is now corrected (L777-780). For all data we applied a statistical test on, sample size was $n = 3$ per treatment group. For such small sample sizes, non-parametric tests are not appropriate due to their severe lack of power. For example, the Wilcoxon rank sum test outputs a minimum p-value of 0.1 for $n = 3$, even if the compared samples, as in our case, are extremely different. We therefore opted to use t-tests.

E) Statistical tests appear mostly at the end of each figure legend. I would prefer to read the statistics in the main text.

Response 7: The revised version of our manuscript contains abridged statistical test results in the main text for all relevant comparisons (L198, L251-252, L337, L342).

F) In lines 242-243, the authors tested the difference between fig 2f and fig 4c, but the corresponding result is "hidden" at the bottom of the legend of figure 4.

Response 8: See Response 7, now added to the main text.

By the way, at the bottom of the legend of figure 4 should be "Fig. 2f and Fig. 4c", not "Fig. 2c and Fig. 4f".

Response 9: Thank you, this is now corrected.

G) The experiments are very clear. If possible, I would like to know better the systems used in the following sense: What is the Inc group of the plasmid encoding the toxin? Also, do the authors know what the helper plasmid is? If the authors have these data, including this information in the Methods section would be useful.

Response 10: We are glad to hear that the descriptions of our experiments are clear. The small plasmid encoding the toxin (pColE2-P9) is not assigned to any particularly incompatibility (Inc) group by the literature or our own analyses: using the PlasmidFinder tool (for the identification and typing of plasmid replicons) on a number of small colicin plasmids including the one used in our study yields a mixture of unspecified “rep_cluster_XXXX” (X being various numbers) assignments. The helper plasmid is the broad host-range, conjugative plasmid R751. We include this information in the Results (L115-117) and at various points in the Methods section (e.g. L680-681, L691).

H) Figures are fine, but Figs 1(d-f) or 3(d-f), etc, are not very clear. For example, in Figs 3e or 3f, it is unclear how green they are (is it closer to 0.5 or 1?).

Response 11: We thank the reviewer for this comment. We use these heat maps intentionally to demonstrate that two key parameters (conjugation rate and toxin potency) which one might reasonably assume will have large implications on the frequency of transconjugants in the system, to show that they actually have very little impact. The important comparison to make in this case is between Fig. 1f and Fig. 3f, which perhaps is not clear to all readers. We have added two new supplemental figures (Fig. S8 and Fig. S9) which we hope demonstrates a starker contrast between the frequency of transconjugants observed in the original model (no metabolic diversity) and the model which includes metabolic diversity.

Minor points:

- 1 - Lines 32-34: I have some problems with the sentence "Genes encoding the production of bacteriocins, antibiotics, and even complex weapons like the contractile type VI secretion machinery, can be horizontally transmitted between cells via transformation (6), conjugation (7-15) and transduction (16,17).". Genes encoding "everything" can be horizontally transmitted between cells via transformation, conjugation and transduction. This is not something special about toxin/immunity genes. I suggest to the authors to change this sentence.

Response 12: We are now more specific here that we are referring to cases where there is evidence that a given weapon can be transferred by starting the sentence as follows:

"***There is evidence that*** *genes encoding the production of bacteriocins, antibiotics, and even complex weapons like the contractile type VI secretion machinery, can be horizontally transmitted between cells via transformation (6), conjugation (7–15) or transduction (16,17).*" (L34-36)

- 2- Line 245 phrasing: "abundances OF the attacker"?

Response 13: The key phrase here is "equal (abundances) to sth.". We have rephrased the relevant sentence to clarify:

"... are now able to reach abundances ***equal to*** *the attacker by the end of the competition ..."* (L253-254)

- 3 - Line 274 "Collectively, these results support our model's prediction that access to a private nutrient can empower transconjugants and promote their invasion into a population of toxin plasmid donors.". Could the authors clarify the meaning of "invasion" here, or clarify the sentence?

Response 14: We now state our intended meaning of invasion more explicitly when we first use it in the introduction as follows (additions highlighted in bold):

"*However, introducing realistic metabolic diversity between strains can greatly amplify the ecological success of transconjugants,* ***enabling the transconjugant strain to rapidly invade a population of the original attacker strain and go from low to high abundance.***" (L59-62)

- 4- Line 365: delete the "," after "could not use"

Response 15: Done.

- 5- Line 368-369: I did not understand this sentence.

Response 16: We have rephrased this as follows:

"*These impacts were particularly strong when the evolution of de novo resistance to the weapon was costly (Fig. 5). There,* ***weapon gene transfer allowed the recipient strain to survive and establish where it would otherwise have gone locally extinct***." (L377-379)

- 6-Lines 416-8: rephrase

Response 17: We have now rephrased this section to read (additions highlighted in bold):

“*Proliferation of transconjugants only occurs under conditions when this proliferation has little or no impact on the abundance of attackers (e.g., compare Fig. 5c and 5d).* ***The result is that there may be little evolutionary benefit for a plasmid-carrying attacker to limit plasmid mobility, because it only leads to the outgrowth of transconjugants that do not compete strongly with the donor for nutrients. Plasmid-borne weapon genes, on the other hand, can clearly benefit from horizontal mobility.*** *While there may be strong natural selection on weapon genes to be mobile, therefore, natural selection on hosts to limit this mobility is likely to be much weaker, which may limit the evolution of host counter-adaptations.*” (L431-438)

- 7- There is a problem with Table S1 (formatting)

Response 18: Apologies. This is fixed in the revised version of the Supplementary Material.

Reviewer #3

This manuscript describes a study to test the hypothesis that conjugation of a plasmid carrying a toxin may change the dynamics of antagonistic interactions between two bacterial populations that are dominated by the antagonism associated with the focal toxin. First, a model assesses the hypothesis relative to a scenario without transfer and a scenario where there is transfer but no toxin. Second, the hypothesis is tested experimentally, showing that conjugation makes relatively little impact in the dynamics. A more complex model allowing for different niches for the bacteria (specific sources of nutrients) suggests an increased impact of the transfer, which is confirmed experimentally. This suggests that horizontal gene transfer of toxins (which is frequent) can impact competition at the ecological scale, which is an interesting and important result. The text is clear and figures excellent (even if legends could be improved).

Response 19: Many thanks for this positive assessment.

Major comments.

While the experimental part is very much developed, the modeling part seems insufficient to sustain some of the statements. There are several issues.

- The model is a standard differential equations model, but experiences are done in a somewhat structured environment, which may affect its applicability.

Response 20: Thank you for this comment. We address this comment and the following two comments in a single response (21) below.

- There are several claims in that the model shows some events are too rare or too frequent. However, there seems to be no clear biological parametrization of the model, i.e. the parameters are not reflecting real conjugation rates, real costs of producing the

toxin, etc. Under these conditions, the model can't produce quantitatively relevant predictions (only qualitative).

- A more thorough exploration of the results of the model when varying the parameters seems essential, especially given that parameters were not obtained from experimental data.

Response 21: We thank the reviewer for these thoughtful comments. Indeed, this model is not parameterized with experimental data. Our aim with the model was to use a simplified system to try to understand general principles about how HGT can influence toxin-mediated competition, rather than try to develop a model which recapitulates experimental conditions. That said, the benefit of a general model is that it can explore very broad parameter space. We agree with your comment that a further exploration of parameter space is useful. A key message we wish to convey with the models is the contrast between transconjugant frequency observed in a scenario in which there is metabolic diversity between interacting strains as opposed to when there is not this metabolic diversity. To highlight this contrast and that the differences seen are robust across parameter space, we performed additional parameter sweeps and provide two new modeling figures which explore parameter space while also placing the two modeling scenarios side by side (Fig. S8 and Fig. S9). We now state this explicitly in the results as follows:

"*The importance of metabolic diversity for our predictions is robust to parameter sweeps that vary the initial density and initial frequency of the attacker and target strains (Fig. S8, S9).*" (L231-233)

- The model seems to start with very small populations that grow to stationary phase. If the goal is to mimic experimental conditions, this is fine. But in real life the populations of residents are probably high and there may be a steady state of growth. It is not clear to me that the results for the invaders stand when residents are both frequent and initially abundant.

Response 22: We thank the reviewer for this comment. As just discussed, to address this we have created a new supplementary figure (Fig. S8) which varies the initial density and initial frequency of the two strains which start each interaction (attacker and target). Changing these initial conditions does not have a major impact on transconjugant frequency nor affect our key conclusions, though they do influence the final frequency of the attacker and the target strains as expected.

One point that should be mentioned, maybe even modeled given the team's experience in the field, is the impact of environmental structure (that can dramatically change this type of dynamics). Of note, these plasmids cannot conjugate in liquid medium. The efficiency of the toxin (or at least its impact on the growth kinetics) may also vary depending on environmental structure.

Response 23: We agree that spatial structure can affect the potential for HGT and other processes and explicit models of spatial structure are an interesting potential avenue for future work. However, despite the fact that modelling did not explicitly capture structure, we were pleased to find that the predictions of the model were supported by our experiments where there was structure, which suggests that our modelling decisions were appropriate for the problem at hand and will not benefit from additional complexity. We now make this clearer in the set up to the experiments as follows:

"*For simplicity, our model assumes that cells are well-mixed in a given patch. However, our experiments introduce a degree of local spatial structure by studying bacteria growing on agar. As we will show, our modelling is robust to this introduction of spatial structure with predictions that are well supported by the experiments.*" (L117-120)

Another point, which in this case may be too complex to add to models and experiences but would be important to discuss, relates with community composition. Many recent works about antibiotic resistance or resistance to phages have shown that results obtained in models or experiences using bacterial communities reveal completely different dynamics from those using clonal minimal systems (as done here). There are several arguments that could apply to this study: the cost of resistance (discussed here in another context), the cost of production (in face of competitors that may be immune since colicins have narrow ranges), the dilution effect that may affect both conjugation (briefly discussed) and killing.

Response 24: We agree that exploring the effects of community composition on the extent and consequences of weapon gene mobility could be an interesting avenue for future studies. We now address this more explicitly in the Discussion:

"*In healthy mammalian guts, E. coli and other Enterobacteriaceae typically only occur at very low densities (48,64–67), which is expected to impede plasmid-driven HGT since it requires direct cell-cell contact. More generally, in diverse communities of bacteria, there is the potential for 'social insulation' whereby third-party species reduce the potential for interactions between strains of a focal species (68,69).*" (L383-387)

Minor comments

- While figures are very clear, some legends are not. They are long, confusing and they don't always clearly describe each panel. They deserve editing. For example legend of Figure 2.

Response 25: We went through and edited the legends to remove words and hopefully improve clarity.

- Line 33. I suggest rephrasing. The sentence suggests that type VI secretion systems can be transferred by transformation or transduction, which has not been shown to the best of my knowledge (the cited references are about effectors not the system).

Response 26: The reviewer is correct. We have rephrased the relevant section to now read:

"*There is evidence that genes encoding the production of bacteriocins, antibiotics, and even complex weapons like the contractile type VI secretion machinery, can be horizontally transmitted between cells via transformation (6), conjugation (7–15)* ***or*** *transduction (16,17).*" (L34-36)

- Line 104. Some colicins are chromosomal (Poey, AAC, 2006)

Response 27: The cited reference concerns the presence of *microcins* on *E. coli* chromosomes. Due to their typically much smaller sizes (<10kDa for microcins, compared to ~40-80kDa for colicins) and in line with recent literature (https://pmc.ncbi.nlm.nih.gov/articles/PMC9517006/), we consider them as different to colicins. To the best of our knowledge, colicins are exclusively encoded on plasmids in *E. coli* and would therefore keep the existing phrasing (L106-107) in the revised version of our manuscript. But also, see below (Response 29) for how we integrated microcins in the revised Discussion.

- Line 373. Low density of E coli may affect conjugation, but also killing by the colicin, which may be neutralized in non-susceptible bacteria?

Response 28: *In vivo,* non-susceptible bacteria are most likely to be either i) *Enterobacteriaceae* with a mutated colicin receptor or some other "resistance" trait (e.g. O-antigens https://pubmed.ncbi.nlm.nih.gov/30510143/), or ii) from a completely different phylum. In both cases, they most likely will not bind the colicin and therefore cannot neutralize it. Non-producing cells expressing the cognate immunity protein can indeed neutralize colicins and reduce killing (https://pubmed.ncbi.nlm.nih.gov/33558498/), but those would most likely be other *E. coli* and would therefore only be present at low densities in the gut, severely dampening any possible neutralization effects.

- Line 397. See comment above about colicins in chromosomes. Also, it's not clear what is the intent of this statement. Why would E. coli be different from the others? And in the chromosomes, aren't the bacteriocins also in MGEs like ICE?

Response 29: As outlined above (Response 27), we consider the colicins different from the microcins, and to the best of our knowledge there is no evidence for colicin genes on *E. coli* chromosomes. As to the purpose of this statement, the total absence of colicin genes on chromosomes makes this class of bacteriocins different from many other well-studied bacteriocins in other species (L410-412), which is why we believe it is worth highlighting.

Regarding chromosomally-encoded bacteriocins (in *E. coli*, only the microcins), the reviewer is correct in stating that these are sometimes associated with MGEs. While many microcins are encoded on plasmids, those that are encoded on the chromosome are often associated with

transposable elements, suggesting that mobility also plays a pivotal role in the evolution of microcins. We have added the following section to the relevant paragraph in the Discussion:

"Interestingly, a different group of bacteriocins produced by E. coli – the microcins – are also strongly associated with gene mobility, as many of them are encoded on plasmids (29,82), and those encoded on chromosomes are frequently associated with transposable elements (83-85). This further supports the view that, in Enterobacteriaceae, genes encoding protein toxins generally benefit from horizontal transmission." (L418-422)

- Line 414 and next. This part of the text is confusing and deserves re-writing.

Response 30: We apologize for the confusion. We have rewritten the relevant section and hope it is clearer now.

New version (additions highlighted in bold):

"*Proliferation of transconjugants only occurs under conditions when this proliferation has little or no impact on the abundance of attackers (e.g., compare Fig. 5c and 5d).* ***The result is that there may be little evolutionary benefit for a plasmid-carrying attacker to limit plasmid mobility, because it only leads to the outgrowth of transconjugants that do not compete strongly with the donor for nutrients. Plasmid-borne weapon genes, on the other hand, can clearly benefit from horizontal mobility.*** *While there may be strong natural selection on weapon genes to be mobile, therefore, natural selection on hosts to limit this mobility is likely to be much weaker, which may limit the evolution of host counter-adaptations.*" (L431-438)

- Line 462. This is incorrect, the literature does not demonstrate that conjugative plasmids always transfer at much higher rates than cognate mobilizable elements. Several examples have been published of similar or close rates (see Charneco, Mic Biotech, 24 or Gaissmaier, Jbac, 22 for recent ones). In some cases the rates of mobilization of the mobilizable element are much higher than those of the conjugative (see Murányi, NAR, 24). Note that many experimental works in the field use (the same) extremely active conjugative plasmids, which creates a bias. (there may also be a confusion here between mobilization and facilitation, as defined in reference 88)

Response 31: We agree that the evidence in the literature supports highly variable conjugation rates for mobilizable plasmids, including much higher, much lower, and similar to conjugative plasmids. While our original phrasing ("typically [...] larger") did not directly contradict this, we agree that it might not accurately reflect the large diversity of possible relative conjugation rates, as well the bias/skew introduced by extremely active conjugative plasmids. We have removed the statement from the relevant Methods section (L475-482).

- It would help to have units in the parameters of Table 1.

Response 32: We thank the reviewer for this comment. We have added units to the parameters listed in Table 1.

- In my pdf of table S1 there are several "Error! Bookmark not defined." needing correction

Response 33: Apologies. This is fixed in the revised version of the Supplementary Material.
